# Paediatric Septic Arthritis of the Hip and the Efficacy of Kocher’s Criteria: A Literature Review

**DOI:** 10.7759/cureus.66184

**Published:** 2024-08-05

**Authors:** Joseph Hanna, Rahel Rashid, Mark Hanna, Beshoy Effat Elkomos, Vikesh Bahadoor, Guirgis Ebeidallah

**Affiliations:** 1 Trauma and Orthopedics, Wirral University Hospital, Cheshire, GBR; 2 General and Colorectal Surgery, Arrowe Park Hospital, Wirral, GBR; 3 Vascular Surgery, Countess of Chester Hospital, Chester, GBR; 4 General and Emergency Surgery, Royal United Hospital, Bath, GBR; 5 Trauma and Orthopedics, Wirral University Teaching Hospital, Wirral, GBR; 6 Emergency Medicine, Salford Royal NHS Foundation Trust, Salford, GBR

**Keywords:** pediatric acute hip pain, pediatric septic arthritis of the hip, transient synovitis of hip, kocher criteria, septic arthritis of hip

## Abstract

Pediatric septic arthritis (SA), an intra-articular infection in children, is considered a surgical emergency. The most commonly affected joints are the lower limb joints. It is more common in children below five years old and in males. Several scoring systems aid in the prediction of the disease and help differentiate it from similar differential diagnoses (such as transient synovitis (TS)). The first and most famous scoring system is Kocher's Criteria (KC), which utilizes a mixture of clinical signs, symptoms, and laboratory markers to predict the likelihood of the diagnosis.

This review aims to assess the current literature to look at primary papers comparing the predicted probability of KC to the original probability described therefore evaluating its efficacy and usefulness in today’s pediatric population. PubMed was searched using the terms “septic arthritis AND hip AND (Kocher OR Kocher’s criteria),” 27 studies resulted, and each study was screened by reading the abstracts. Six studies were included in this review. Inclusion criteria were any study that looked at SA of the hip in the pediatric population prospectively or retrospectively, using KC to help make a diagnosis and looking at the predicted probability of KC. Exclusion criteria included studies looking at adults, joints other than the hip, and papers not assessing the predicted probability.

The efficacy of KC for diagnosing SA is not well-supported by current literature. Studies indicate that KC have low specificity for SA, suggesting it should not replace arthrocentesis as the diagnostic gold standard. Clinicians should use this model cautiously, and more extensive, prospective studies are needed to validate its effectiveness.

## Introduction and background

Septic arthritis (SA) is defined as an intra-articular infection or an infection of a joint [1]. Epidemiological studies show that the incidence of this disease ranges from five to 37.1/100,000 per year making it a rare but important disease [2,3]. The most commonly affected joints are lower limb joints accounting for 80% of cases, with 35%-40% of cases involving the hip [4]. SA is more common in males than females (3:2 ratio) and in children under five [5]. Other risk factors for developing SA include previous trauma as well as conditions instigating poor immune function such as diabetes, HIV, and drug and alcohol abuse [5,6].

Pathophysiology and clinical features

The etiology of SA can be divided into three main categories. (1) Direct inoculation through trauma or iatrogenic causes such as surgery or injections. (2) Extension from adjacent infected bone (osteomyelitis). (3) Most commonly via hematogenous spread following respiratory tract infections [7,8]. The most common bacterial organism causing SA is Staphylococcus aureus [9,10], usually in its methicillin-sensitive form [11,12]. Other organisms include Group A and B Streptococcus, Neisseria gonorrhoeae, and Haemophilus species, Aggregatibacter actinomycetemcomitans, Cardiobacterium hominis, Eikenella corrodens, and Kingella kingae (HACEK) organisms of which K. kingae is seen more frequently in children less than four years old [4,10,13].

Once the bacteria invade the synovium due to a lack of basement membrane, it occupies the joint space. Once in the joint space, the body begins to initiate an inflammatory process. Inflammatory cytokines and proteases (specifically interleukin-1, interleukin-6, and tumor necrosis factor) cause the upregulation of “toll-like receptor (TLR)” pathways. As well as this, the inflammatory response triggers an influx of macrophages, B cells, and T cells. All the above inflammatory cells instigate joint destruction, specifically to the vasculature and the articular cartilage [14]. Furthermore, bacterial toxins and microbial surface components promote the binding of bacteria to intra-articular proteins/the joint matrix, further enhancing the damage to the joint and aiding in fulminating the infection [14].

Clinical features of SA include fever, monoarthritis (only one joint is commonly affected), inability to weight bear, restricted range of movement, and limping in children. Signs may include fever, warmth, redness, and effusion of the joint [1,3,4]. There are several alternative similar differential diagnoses such as transient synovitis (TS), juvenile idiopathic arthritis, and even trauma. Ensuring a prompt and correct diagnosis is essential given the complications that may occur if misdiagnosed or there is a delay in diagnosis [15].

Diagnosis

A high index of clinical suspicion is required to make a diagnosis, this is aided by imaging, blood tests, and several predictive scoring systems. Imaging includes plain radiographs with antero-posterior (AP) and frog-leg lateral pelvic views as well as Point-of-Care Ultrasound (POCUS) assessing the presence of an effusion [16-18]. MRI has also been recently utilized in both diagnosing and monitoring effusions following surgical intervention with greater accuracy than both plain radiographs and POCUS [19,20]. Laboratory serum blood investigations include infection and inflammatory markers, more specifically the three most utilized markers are white cell count (WCC), C-reactive protein (CRP), and Erythrocyte sedimentation rate (ESR) [21,22].

There are several scoring systems utilized to predict the disease and help differentiate it from similar differential diagnoses such as TS. The first and most famous scoring system is Kocher’s criteria (KC) which utilizes a mixture of clinical signs and symptoms as well as the laboratory markers mentioned above to predict the likelihood of the diagnosis. Professor Kocher was an American Orthopedic surgeon, who published paper in 1999 describing a predicted algorithm that would be useful in differentiating SA of the hip in children from TS in the same cohort. This algorithm was later known as “The Kocher’s criteria” [23].

The above diagnostic tests of history, examination findings, imaging, blood tests, and scoring systems help formulate the probability of the diagnosis; they are not diagnostic. The gold standard diagnostic test is arthrocentesis or hip aspiration. Aspiration allows the fluid to be sent for culturing so antibiotics can be specifically chosen based on culture and sensitivity results.

Treatment

As mentioned previously, SA is a surgical emergency and prompt diagnosis and treatment are essential to avoid complications, morbidity, and even mortality. The options for treatment include operative and non-operative management [1,24]. The majority of septic hip arthritis requires operative management with surgical incision and drainage being the mainstay of treatment. This reduces intraarticular pressure and decreases epiphyseal ischemia. Long-term antibiotics based on culture and sensitivity results are also used alongside operative intervention [8,15]. Non-operative management is hardly used, indications for this may be in adolescent Neisseria gonorrhoeae infection where antibiotic treatment may be sufficient.

A recent paper by Wang et al. describes novel treatment modalities within the literature that may be beneficial in the future, especially for bacteria resistant to several antibiotics such as methicillin-resistant S. aureus (MRSA). These new modalities are non-antibiotic based and focus on downregulating the immune response described above in the description of pathophysiology. These include “matrix metalloproteinases (MMPs)” based therapy as well as several interleukin blockage therapies. However, these have only been established in various in vitro and non-human, model-based studies and therefore require further research ideally in the form of randomized controlled trials [15].

## Review

Methodology

PubMed was searched using the terms “septic arthritis AND hip AND (Kocher OR Kocher’s criteria).” Twenty-seven studies were conducted, and each study was screened by manually reading the studies. Inclusion criteria were any study that looked at the pediatric population prospectively or retrospectively, using KC to help make a diagnosis and looking at the predicted probability of KC. Exclusion criteria included studies looking at adults, joints other than the hip, and papers not assessing the predicted probability of KC. Six studies were included in this review.

Results

The six studies were published from 1999 to 2023 and looked at data ranging from four years to 17 years. These included Kocher’s original paper in 1999 and his own revalidation in 2004. Four of the six studies were retrospective whilst Kocher’s revalidation and the study by Caird et al. were both prospective. The characteristics of each study can be seen in Table 1.

**Table 1:** Characteristics of the included studies

Study	Sample size	Study type	Years	Journal
Kocher et al. 1999 [23]	282 patients	Retrospective	17 years	The Journal of Bone and Joint Surgery
Luhmann et al. [25]	263 patients	Retrospective	8 years	The Journal of Bone and Joint Surgery
Kocher’s Revalidation 2004 [26]	213 patients	Prospective	5 years	The Journal of Bone and Joint Surgery
Caird et al. [27]	53 patients	Prospective	4 years	The Journal of Bone and Joint Surgery
Sultan et al. [28]	96 patients	Retrospective	4 years	The Bone and Joint Journal
Olandres et al. [22]	101 patients	Retrospective	5 years	Archives of Orthopaedic and Trauma Surgery

Kocher’s original paper in 1999 was a retrospective analysis of 282 patients over 17 years (1979-1996) who had presented with hip pain. One hundred fourteen of these patients were excluded for various reasons, 86 had a diagnosis of TS and 82 had a diagnosis of SA. Data were obtained from all included patients, starting from the usual history and physical examination, with a particular focus on history of fever, inability to bear weight, recent or current infection, and trauma. Serum WCC, ESR, as well as joint fluid culture and sensitivity. Univariate analysis was conducted to study the significance of each variable, using Fisher's exact test for categorical variables and the two-sample Student t-test for continuous variables.

History of recent antibiotic use, history of chills, gender, ESR above 40, history of fever (above 38.5), inability to weight bear, and serum WCC above 12,000 cells per cubic millimeter (12.0 x 10^9^ cells per liter) were all found to be statistically significant. Following this, the authors used multivariate analysis with multiple logistic regression combining the last four variables discussed above and calculating the predicted probability of SA as the number of positive variables increased. The predicted probability of SA was determined for all 16 combinations of these four predictors. Kocher quoted a predicted probability of 99.6% of SA as opposed to TS if all four variables were present in a patient, 93.1% if three of the variables were present, 40% if two, and 3.0% if one variable was present [23].

Luhmann et al. published a retrospective comparative study looking at eight years’ worth of data. It identified 265 hips that underwent arthrocentesis, of which 165 were included; there were 47 patients diagnosed with SA and 118 with TS. The study showed that if all four of the predictive criteria were present, the predicted probability was only 59%, in contrast to the 99.6% in Kocher’s original paper [25].

Kocher’s revalidation, published in 2004, was a prospective study in a new cohort of patients. Again, both univariate and multivariate analysis were utilized to assess the statistical significance of any variables and then assess predicted probability with the original four variables discussed. Interestingly, they discovered a similar predicted probability to the original study, with a 93% probability in patients having all four variables. They also assessed the sensitivity of KC as well as false positive rates. The sensitivity of the test when all four predictors were present halved in the validation (0.31 vs 0.16). However, specificity was not quoted [26].

Caird et al. published a similar study to Kocher’s revalidation. This was also a prospective study attempting to validate Kocher’s predictive tool and utilized univariate and multivariate analysis to do so. This study prospectively collected data over four years gathering information for 53 patients who underwent hip aspiration. They also introduced CRP as another predictive variable making it five variable criteria. The predicted probability of SA, if four variables were present, was 93.1% and increased to 97.5% if all five variables were positive (including CRP) [27].

Sultan and Hughes published a similar study in 2010 attempting to validate the predictive model. This was a retrospective study carried out in a district general hospital identifying 137 patients presenting with hip pain of which 96 were included. Of these only five had SA, and the remainder had TS. The predicted probability was found to be lower than initially described at 39.4% if four criteria were present, and 59.9% if Caird’s criteria or the modified KC were utilized. This study also considered the sensitivity and specificity as the number of positive variables increased. The specificity was only 0.516 when only one predictive criterion was positive [28].

Olandres et al. published the most recent study in 2023; this was a retrospective study attempting to assess the sensitivity and specificity of a combination of raised CRP and USS finding of effusion in diagnosing SA compared to TS. Their cohort had seven of the 101 patients that were diagnosed with SA. Although this study did not directly validate KC, it did look at the predicted probability using the modified Kocher’s five suggested variables and compared this to the novel technique of CRP and USS. It quoted a probability of only 59.16% if Kocher’s four clinical predictors were present, this went up to 87.81% if the five criteria were present [22]. The published predicted probabilities with an increasing number of variables can be seen in Table 2, this can also be compared easily with the succeeding literature.

**Table 2:** Comparison of the predicted probability at each stage of predicted criteria in the studies N/A: Not available - was not measured in the original study

Study	0 predicted criteria - Predicted probability (%)	1 predicted criteria - Predicted probability (%)	2 predicted criteria - Predicted probability (%)	3 predicted criteria - Predicted probability (%)	4 predicted criteria - Predicted probability (%)	5 predicted criteria - Predicted probability (%)
Kocher et al. 1999 [23]	<0.2	3.0	40.0	93.1	99.6	
Luhmann et al. [25]	N/A	N/A	N/A	N/A	59.1	
Kocher’s Revalidation 2004 [26]	2.0	9.5	35	72.8	93	
Caird et al. [27]	16.9	36.7	62.4	82.6	93.1	97.5
Sultan et al. [28]	2.3	5.1	10.9	22.0	39.4	59.9
Olandres et al. [22]	0.24	1.16	5.53	22.55	59.16	87.81

Discussion

Differentiating between TS and SA of the hip in children can be challenging, yet crucial. Not only do they have different underlying pathophysiology and treatment strategies, but SA is considered a surgical emergency and can cause serious complications. There is no simple or effective test to help distinguish between the two. That’s why clinicians consider a host of signs and symptoms from history, physical examination, and laboratory values. Predictive models are usually developed to face such challenges. The first of such predictive models was KC.

Kocher’s original 1999 paper was a retrospective comparative study that looked at 282 patients presenting with hip pain. Of those 114 were excluded, 82 had a diagnosis of SA, and 86 had a diagnosis of TS. Several blood results, clinical signs, and characteristics were recorded for each patient included in the study; univariate analysis was utilized to assess the statistical significance of each variable in differentiating SA from TS. Kocher identified four key factors: the presence of fever (defined as oral temperature >38.5 degrees Celsius), inability to bear weight on the affected side, elevated serum WCC (>12.0 x 10^9^ cells/L), and ESR (>40 mm/hr). Kocher reported a 99.6% predicted probability of SA if the patient had all four factors present [23]. Soon after its publication, multiple attempts were made to revalidate the KC, with conflicting results.

Three of the six included studies showed strong predicted probabilities when four of the predicted criteria were met. These papers included Kocher’s original paper in 1999, his revalidation in 2004, and Caird et al. in 2006. Although the results published by Kocher et al. in the original paper in 1999 were promising and showed good, predicted probability, it is important to understand that clinical predictor models can often be unreliable in a new patient population as they tend to be more optimally modeled to the original data set [23,26,27].

In Kocher’s revalidation, published in 2004, the authors attempted to validate the predictive model by using it in a new cohort of patients prospectively. Univariate and multivariate analyses were utilized to assess the statistical significance of any variables and then assess predicted probability with the original four variables discussed. Interestingly, they discovered a similar predicted probability to the original study, with a 93% probability in patients having all four variables. They also assessed the sensitivity of KC as well as false positive rates. The sensitivity of the test when all four predictors were present halved in the validation (0.31 vs 0.16). Although one could argue this is more important for a diagnostic test such as this, ruling out SA is much more significant. Given, that this was published in the same hospital by the same authors, there may also be some degree of unintentional bias.

Caird et al. published a similar study to Kocher’s revalidation. This was also a prospective study attempting to validate Kocher’s predictive tool and utilizes univariate and multivariate analysis. This study is the first to introduce CRP as another predictive variable making it five variable criteria, this is commonly now known as “the modified Kocher’s criteria.” Again, the results were promising as the predicted probability of SA, if four variables were present, was 93.1% and increased to 97.5% if all five variables were positive (including CRP). Unfortunately, this paper also has some limitations. They only looked at children in whom the findings were so suspicious for SA that hip aspiration was performed. This means this cohort of patients was already clinically assumed to be at high risk of SA whilst the KC was initially intended to be used for all patients presenting with atraumatic hip pain where SA or TS was the differential diagnosis. Another limitation of the study is its sample size; a total of 53 patients undergoing hip aspiration is not a very large sample size.

On the other hand, the other three studies, included in this review, found the predicted probability to be much lower than initially described by Kocher et al. Luhmann et al. published the first paper (in 2004) attempting to validate Kocher’s predictive model. This was a retrospective comparative study looking at eight years’ worth of data. This study showed that if all four of the predictive criteria were present, the predicted probability was only 59%, in contrast to the 99.6% in Kocher’s original paper. Again, this study is limited in that it looked at patients who underwent hip aspiration. Although this is not the intended cohort of patients, it actually should have a higher predicted probability (similar to Caird’s) as these patients were clinically felt to have SA. Another limitation of this study is that it only looked at predicted probability when all four criteria were present.

Sultan published a similar study in 2010 attempting to validate the predictive model. This was a retrospective study carried out in a district general hospital identifying 137 patients presenting with hip pain of which 96 were included. Of these only five had SA, and the remainder had TS. Again, the predicted probability was found to be lower than initially described at 39.4% if four criteria were present and 59.9% if Caird’s criteria or the modified KC was utilized. This study also considered the sensitivity and specificity as the number of positive variables increased. Unfortunately, the specificity was only 0.516 when only one predictive variable was positive suggesting it was a poor test to rule out SA. This study was mainly limited by its size as only five patients with SA were identified. Although this was used in the intended cohort of patients and is likely more representative of the general population/ published epidemiological data.

Finally, Olandres et al. published the most recent study in 2023, this was also a retrospective study attempting to assess the sensitivity and specificity of a combination of raised CRP and USS finding of effusion in diagnosing SA compared to TS. Although this study did not directly validate KC, it did look at the predicted probability using the modified Kocher’s five suggested variables and compared this to the novel technique of CRP and USS. It quoted a probability of only 59.16% if Kocher’s four clinical predictors were present, this went up to a respectable 87.81% if the five criteria were present. Again, this study was limited by size as only seven of the 101 patients were diagnosed with SA.

## Conclusions

The current literature does not provide sufficient evidence to fully establish the efficacy or usefulness of KC in diagnosing SA. Of the six reviewed studies, three indicated good, predicted probability but were either authored by the original researcher or focused on inappropriate patient cohorts. The other three studies, which showed poor predicted probability, had small sample sizes, compromising their statistical strength. Several studies demonstrated that KC has low specificity for SA, indicating it should not be used to rule out the diagnosis. Clinicians are advised to use this predictive model cautiously and not as a replacement for the diagnostic gold standard of arthrocentesis. Further research with larger, possibly multicentric, prospective studies is necessary to validate this predictive model.
